# Nictitating membrane fixation improves stability of the contact lens on the animal corneal surface

**DOI:** 10.1371/journal.pone.0194795

**Published:** 2018-03-27

**Authors:** J. Jeremy Chae, Yu Jung Shin, Justin D. Lee, Kangmoon Seo, Jennifer H. Elisseeff

**Affiliations:** 1 Translational Tissue Engineering Center, Wilmer Eye Institute and Department of Biomedical Engineering, Johns Hopkins University, Baltimore, Maryland, United States of America; 2 Department of Ophthalmology, Johns Hopkins University, Baltimore, Maryland, United States of America; 3 Department of Veterinary Clinic, College of Veterinary Medicine, Seoul National University, Seoul, Republic of Korea; Central Michigan University College of Medicine, UNITED STATES

## Abstract

We evaluated the feasibility and safety of nictitating membrane fixation to address reduced contact lens stability by the nictitating membrane in a rabbit model. Under general anesthesia, twelve animals received a horizontal mattress suture between the nictitating membrane and the upper eyelid of one eye. To assess the effects of this technique and secondary side effects, contact lens stability test, Schirmer tear test, tear break-up time measurement, eye tissue pathology and morphology were evaluated. Contact lens stability was increased after nictitating membrane fixation. The percentage of contact lens retention in the nictitating membrane fixed rabbit after 4 hours was 90% whereas that in the untreated rabbit was 42.5%. In addition, there were no significant differences in tear quantity and quality between the fixed and untreated eyes. Furthermore, no remarkable pathological lesions were found in gross observation during the 1-month time period or the following pathological examination. In this study, we demonstrated that nictitating membrane fixation increases contact lens stability without specific side effects using a rabbit model. This minimally invasive procedure could be useful when designing animal models for testing new contact lenses and has potential to apply to other biomaterial research on the ocular surface.

## Introduction

Animal models are the primary tool to evaluate comprehensive physiological responses to biomaterials in the ophthalmic research. A proper animal model study is considered an essential procedure to ensure a successful translational application of an ophthalmic biomaterial to clinical use. However, the anatomical and physiological differences between humans and animals can preclude accurately capturing the anatomy and physiology of the human eye [1]. The nictitating membrane (NM), also termed the third eyelid, exemplifies species-specific differences of the eye. Anatomically, most animals have this accessory eyelid around the medial canthus, whereas human and anthropoids keep a vestigial remnant of this organ, *plica semilunaris* [2]. Physiologically, the function of the *plica semilunaris* in human is insignificant, but the NM in animals contributes to a healthy animal eye by producing and distributing tears, removing ocular debris, secreting immune proteins, and acting as a mechanical barrier [3]. The NM can limit animal studies of a new biomaterial, as a soft or a vulnerable material applied on the ocular surface can be easily damaged by movement of the NM. Additionally, if the material weakly binds to the ocular surface, it could be displaced and consequently removed from the animal by NM.

Contact lens application can be hindered by both limitations. Although the NM could help keep a contact lens hydrated, it also reduces stability of the contact lens, preventing further preclinical study [4]. To increase stability, nictitating membranectomy and tarsorrhaphy have been used to remove the membrane or limit movement, respectively. However, excision of NM is an invasive procedure, and could cause mild to moderate dry eye syndrome [5] which leads to pathological cascades on the ocular surface [6] in some species. Closure of the palpebral aperture by suturing both eyelids decreases oxygen levels [7, 8], alters tear fluid osmolality [9] and swells the corneal epithelium [4]. Thus, these surgical procedures cause undesired secondary effects resulting in unreliable biocompatibility data for materials in the eye.

To improve upon existing models for preclinical biomaterials testing in the eye, especially contact lens research, we developed a method to stabilize the nictitating membrane. By fixing the NM to the upper eyelid, the movement of the membrane can be limited which may lead to increased material stability on the ocular surface. We evaluated the feasibility and safety of NM fixation to improve material stability and ultimately biocompatibility testing.

## Materials & methods

### Animals

Twelve New Zealand white male rabbits, 2.0 to 3.5 kg in weight, were used in this study. All experimental procedures in this study accord to the Association for Research in Vision and Ophthalmology (ARVO) Statement for the Use of Animals in Ophthalmic and Visual Research, and were approved by the Institutional Animal Care and Use Committee at Johns Hopkins University. Ten animals were used for tear and contact lens stability tests, and two animals served for gross observation and pathological examination.

### Nictitating membrane fixation

**Fig 1:**
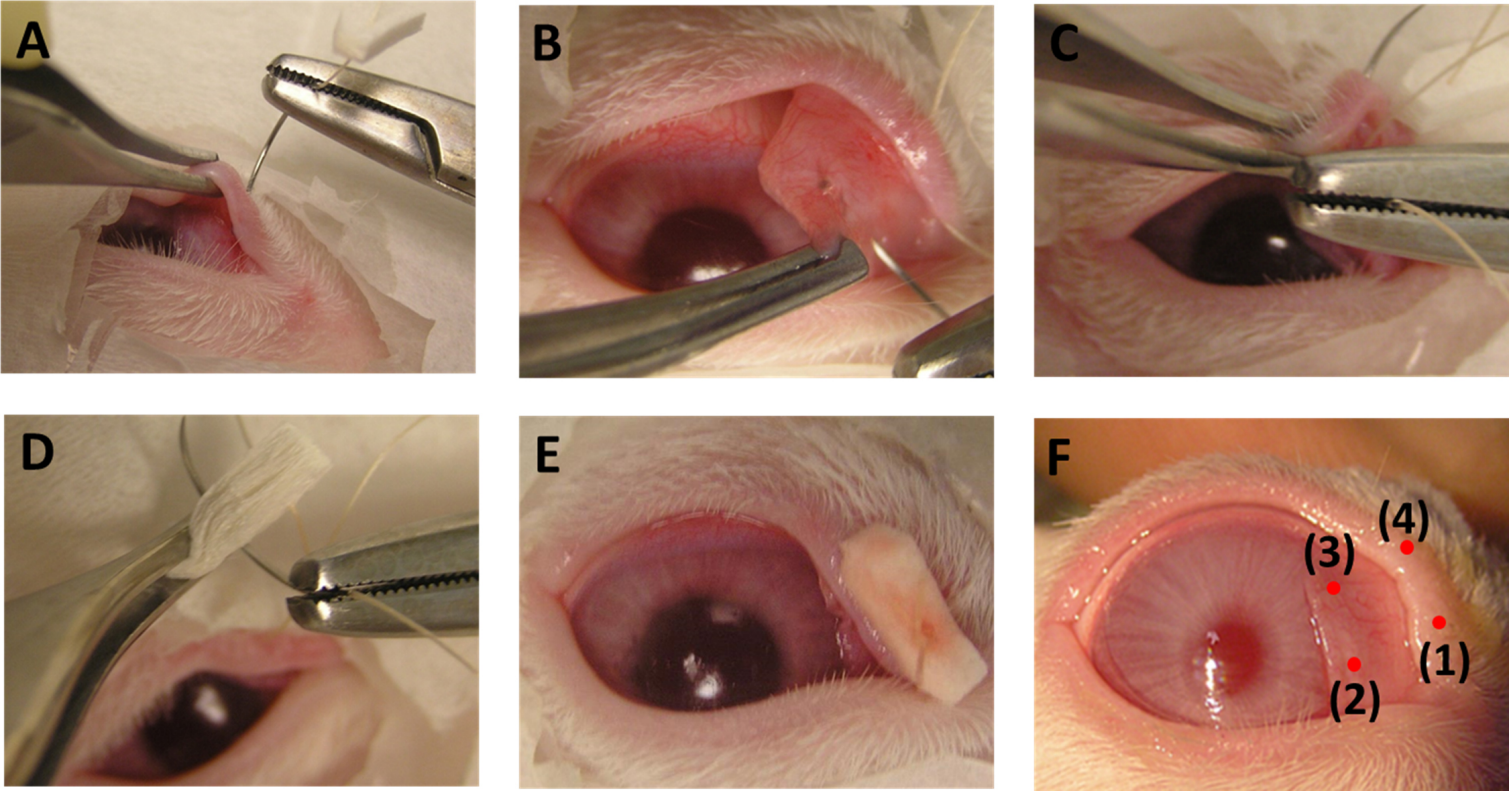
Surgical procedure for nictitating membrane fixation. (A) After penetrating a tailored ophthalmic spear, the needle passed through the upper eyelid and then the palpebral conjunctiva. (B) A stitch was applied on the lateral part of the nictitating membrane. (C) The following bite was back out the palpebral conjunctival and passed through the upper eyelid. (D) Lastly, the needle penetrated the ophthalmic spear and (E) the suture was knotted. (F) Four bitten areas are shown in rabbit eyelid.

Membrane fixation was performed under general anesthesia with ketamine (15 mg/kg of body weight) and xylazine (2 mg/kg of body weight) delivered intramuscularly. NM fixation was applied to a randomly chosen eye and the other eye was untreated to serve as a control. After administering one or two drops of topical anesthesia (proparacaine 0.5%), a horizontal mattress suture using a 4–0 polyglactin 910 (Vicryl, Ethicon, Somerville, NJ) was placed between the free edges of the nictitating membrane and the upper eyelid. The suture needle first penetrated an ophthalmic spear cut 5 (length) x 10 (width) mm in size. Next, the suture was passed the upper eyelid through the palpebral conjunctiva in the medial canthus area (Fig 1A). The last suture was placed about 4–6 mm in length along the lateral margin of nictitating membrane, approximately 2 mm apart (Fig 1B). The following suture bite was backed out the palpebral conjunctival and passed through the upper eyelid (Fig 1C) and the ophthalmic spear (Fig 1D). The suture knot was made on the spear (Fig 1E), and the spear was trimmed to stay within the margin of the eyelid.

### Schirmer tear test and tear break-up time

To assess the change of quantity and quality of tear production after the NM fixation procedure, Schimer tear test-1 (STT-1) and tear break-up time (TBUT) measurements were performed for both eyes (*n =* 10 rabbits) 6 hours after the surgery, and at days 1, 2 and 3. After bending the notch of a STT strip (Alchon®, Fort Worth, TX), the folded end was placed within the inferolateral one-third of the lower conjunctival fornix for 3 minutes. The level of tear migration was marked and measured from the fold, immediately at removal. TBUT was assessed under blue light in a dark room. Each eyelid was closed manually to distribute the tear film. Then, the eye was held open and two drops of sodium fluorescein dye (Fluoresoft -0.35%^®^, Alden optical, Lancaster, NY) were added onto the corneal surface. The time for observing distortion of the reflected image was recorded.

### Contact lens stability test

After the tear tests, rabbits underwent contact lens stability tests. Each contact lens (Hydrokone^®^, Visionary Optics, Front Royal, VA) was confirmed to fit each eye with the conventional contact lens fitting method using florescent dye and a slit lamp microscope. The base curvature and the diameter of lens were 8.4–8.6 and 14.00 mm, respectively. We measured stability of the contact lens by calculating the percentage of “successful retention”, which was defined in this study as the contact lens remaining on the corneal surface for more than four hours after application. The baseline time was selected to prevent over-wearing contact lens for first-time contact lens wearers. The contact lens was applied four times per an animal.

### Gross observation and pathological examination

Two rabbits not used for either tear or contact lens tests were observed for one month after NM fixation. The florescent dye test was performed to ensure health of the corneal surface after NM fixation. Each eye was examined with a conventional slit lamp microscope after the procedure, at days 7, 14 and 30. After 30 days, the rabbits were sacrificed for pathological examination. The tissue was collected from the eye that underwent the procedure and the collateral eye served as a control. Dehydrated and paraffin-embedded sections were stained with hematoxylin and eosin (H&E) using standard techniques. Immunohistochemistry was also conducted on paraffin-embedded sections. After deparaffinization and rehydration, slides were immersed in a solution of sodium citrate (pH 6.0) on a water bath at 95°C for 90 minutes. Then, the sections were incubated overnight at 4°C with one of primary antibodies against CD3 (1:100; Abcam, ab11089), CD4 (1:100; Abcam, ab25804), CD8 (1:50; Santacruz, sc-59117) and beta-III tubulin (1:100; Abcam, ab7751). Next day, the slides were exposed to secondary antibodies including Cy3 goat anti-rat (1:200; Abcam, ab150159, CD3), Alexa Fluor^®^ 647 goat anti-mouse (1:200; Thermo Fisher Scientific, A21235, CD4 and CD8), and Cy3 goat anti-mouse (1/800; Jackson Immunoresearch, beta-III tubulin) for 1 hour at room temperature. Finally, the nuclei were counterstained using1 μg/mL DAPI (4′,6-diamidino-2-phenylindole dihydrochloride; Thermo Fisher Scientific, D1306) for 45 minutes at room temperature.

### Statistical analysis

Data from tear tests (STT values and TBUT) were presented as means ± standard deviation (S.D.). Results were analyzed by Mann-Whitney test to evaluate the differences between two eyes at each time point using SPSS 15.0 for Windows (SPSS Inc., Chicago, IL). Statistical significance was established at *p* < 0.05.

## Results

### Tear tests

**Fig 2:**
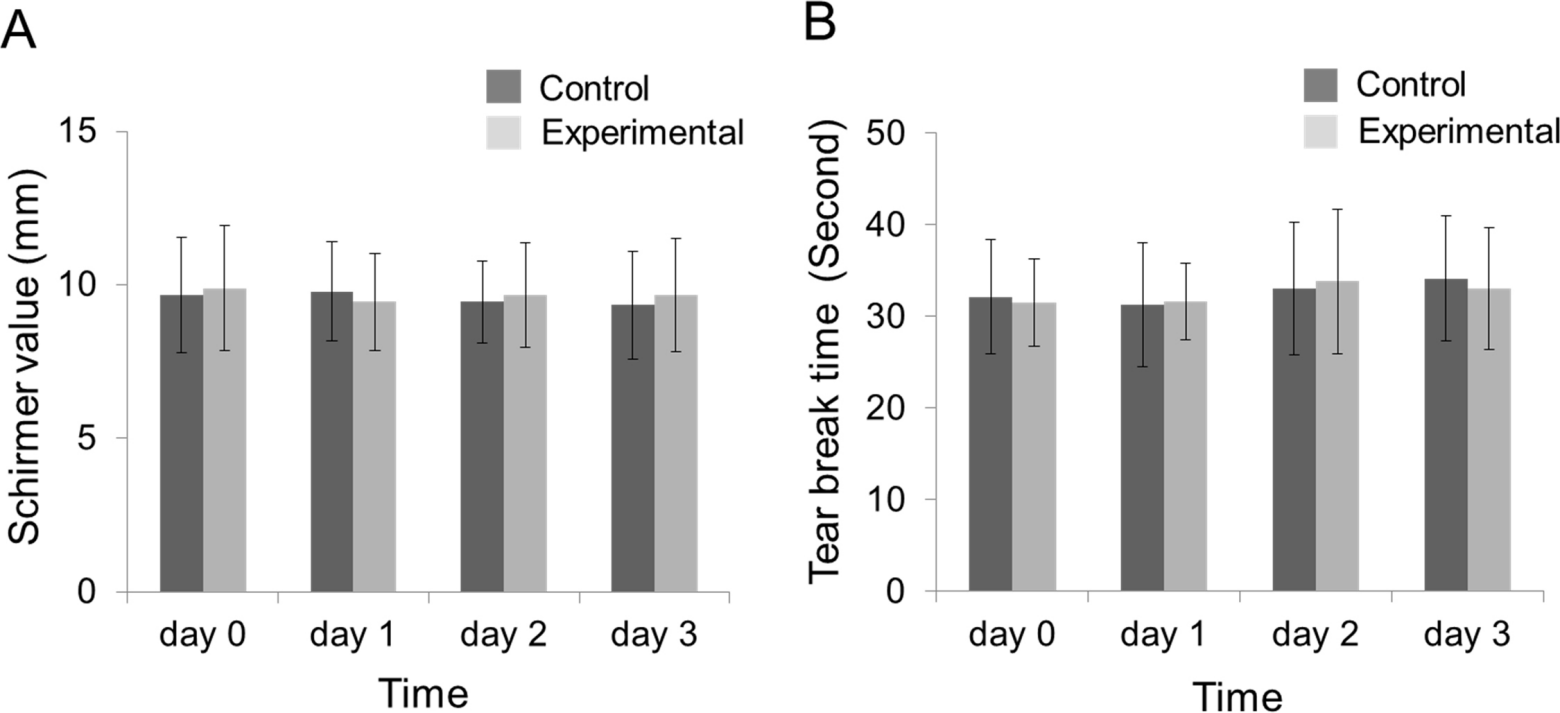
Evaluation of tear quantity and quality. Rabbits underwent nictitating membrane fixation in one eye, while the other eye was left untreated as a control. (A) Shimer tear test (STT). (B) Tear break-up time (TBUT) measurement. Data are averages ± SD (*n* = 10). No significant difference was found between both groups of STT values and TUBT measurements. Statistical significance was evaluated by Mann-Whitney test at *p* < 0.05.

To evaluate secondary effects for the secretary system after NM fixation, we tested tear production quantity and quality. No significant differences of STT values and TBUT measurements were found between the control and experimental eyes. In the fixed eyes, the average STT value was 10.1 ± 2.0 mm 6 hours after the procedure on day 0 (Fig 2A). The values for both eyes were consistent through the three-day experiment, ranging from 9.5 to 10.2 mm. The TBUT in both groups also did not fluctuate significantly over the course of 3 days after NM fixation, ranging from 31.5 to 33.9 seconds (Fig 2B). Thus, the NM fixation procedure did not change the quantity and quality of tear production.

### Contact lens stability test

Both contact lens curvatures of 8.4 and 8.6mm fitted well on the all rabbit corneas. The stability of contact lens was increased after applying NM fixation, where the percentage of contact lens retention—considered to be the lens remaining on corneal surface after 4 hours—in the rabbit eyes that had procedure was higher than untreated eyes (90% versus 42.5%, respectively).

### Gross observation and pathological examination

**Fig 3:**
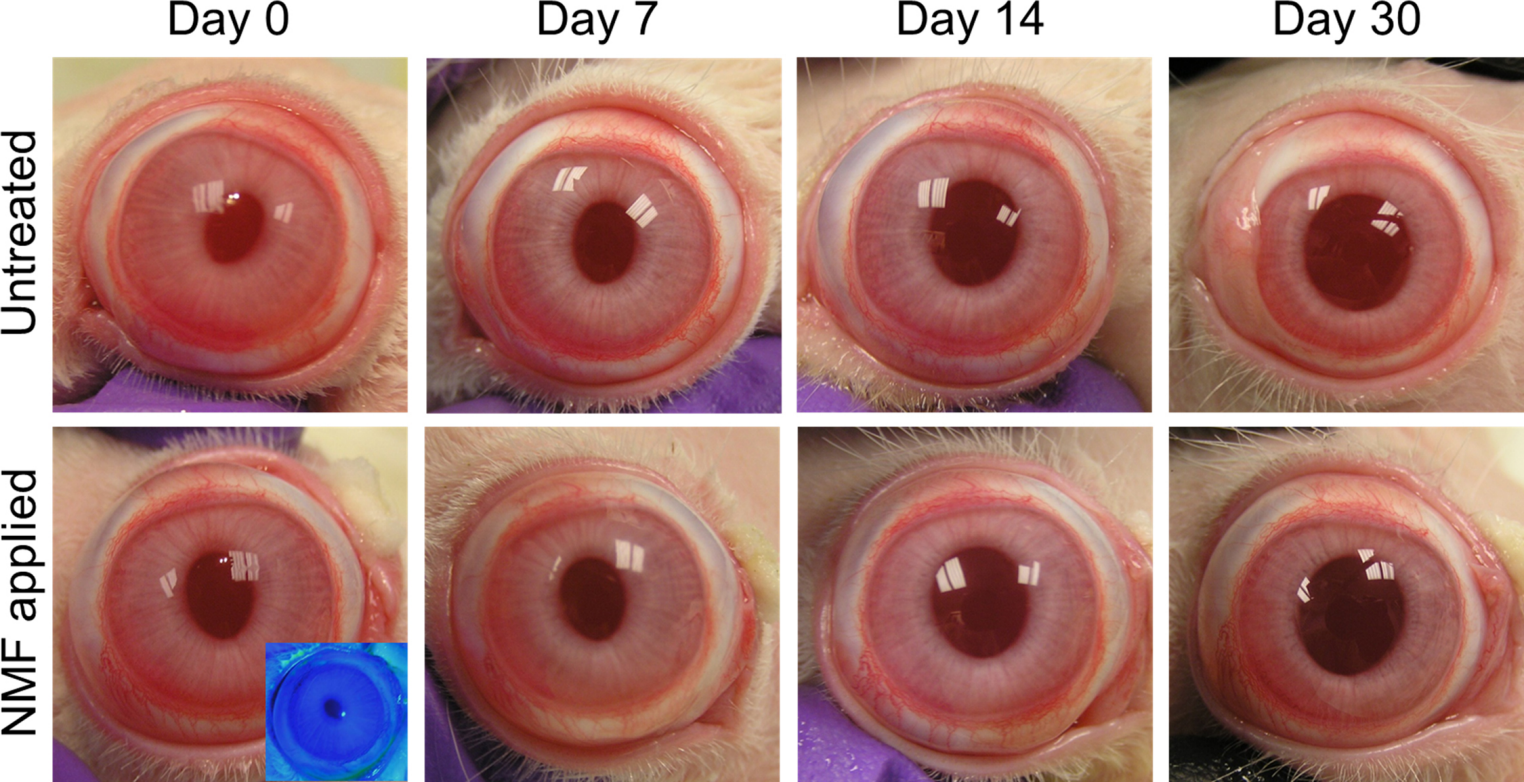
Gross observation for untreated and nictitating membrane fixed eyes. After applying nictitating membrane fixation (NFM), eyes were observed in two rabbits over a 1-month period to look for lesions such as corneal scar and conjunctival redness. There were no substantial lesions in either eyes and there was no procedure-induced damage on the corneas. Images are representative of *n* = 2 rabbits.

**Fig 4:**
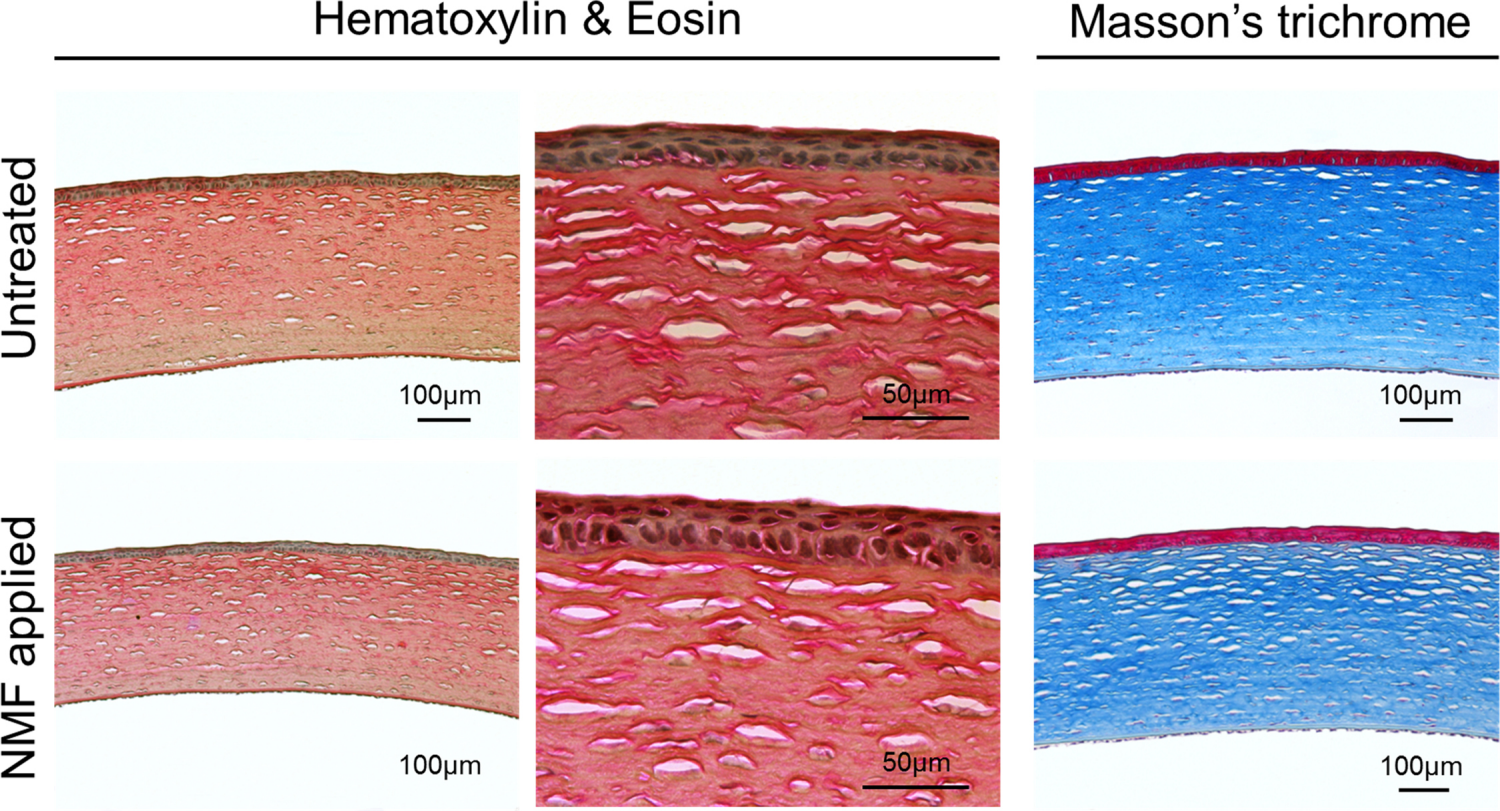
Pathological examination for experimental and control corneas. Corneas were stained at 1 month with H&E and Masson’s trichrome. Corneas from both untreated and nictitating membraned fixation (NMF) applied eyes did not show any remarkable pathological lesions. Images are representative of n = 2 animals.

**Fig 5:**
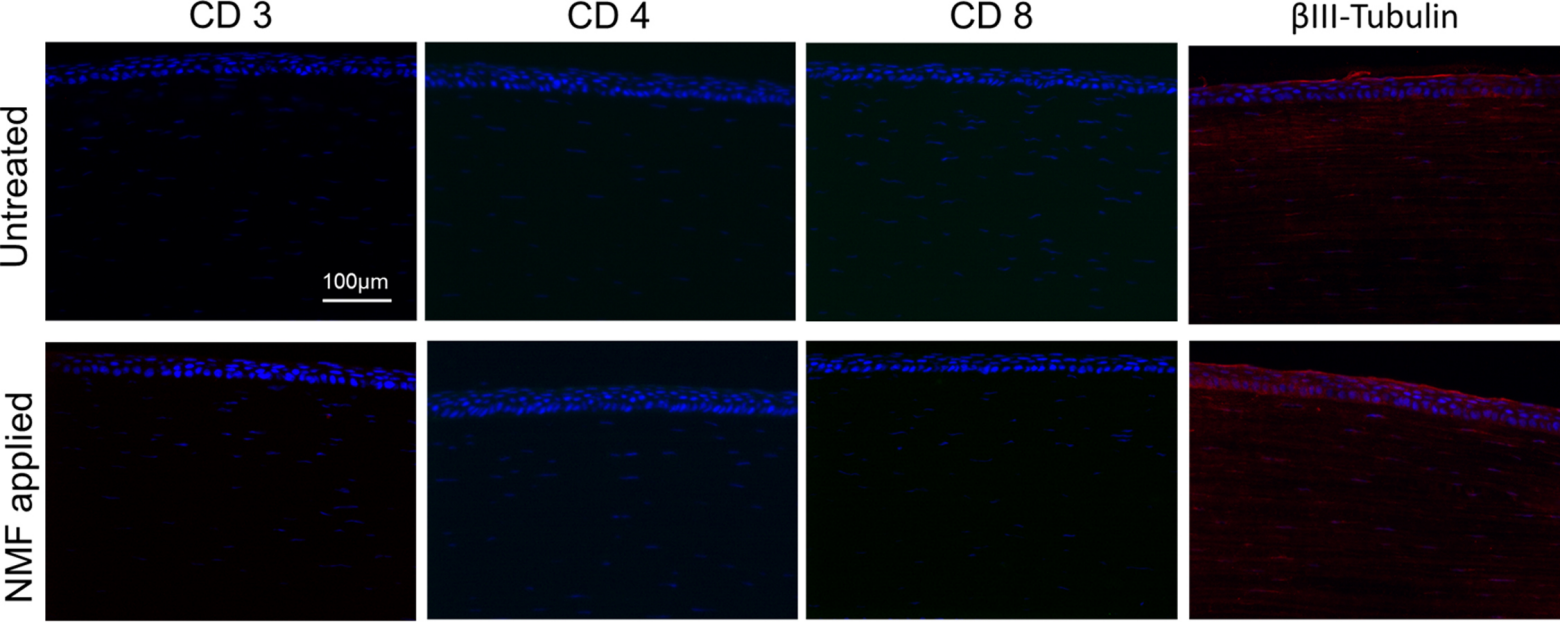
Immunostaining of CD3, CD4, CD8 and βIII-Tubulin for experimental and control corneas. Immunohistochemistry was performed on rabbit corneas using anti-CD3, CD4, CD8 and βIII-Tubulin antibodies 1 month after surgery. Corneas from both control and experimental groups did not present any sign of a deleterious immune responses. Nerves were distributed through the cornea without any areas of degeneration observed in either group. DAPI was used to stain the nuclei (blue). Images are representative of n = 2 animals.

In the florescent dye test, there was no region on the ocular surface that stained after the procedure, confirming that the surface was not damaged (Fig 3). During the course of 1 month after NM fixation, we observed no substantial lesions, including corneal haze, conjunctival hyperemia or chemosis, aqueous flare, or iris lesions in the both untreated and NM-fixed eyes (Fig 3). Normal Purkinje image was found in the both sets of eyes during this time period. Corneas from both untreated and fixed eyes did not show any remarkable pathological lesions upon H&E staining. Additionally, Mason’s trichrome staining confirmed there was no scar tissue formation in either eye (Fig 4). In the immunohistochemistry evaluation, the cornea from both groups did not display any positive staining of immune cell markers including CD3, CD4 and CD8. Another marker, beta-III tubulin, demonstrated that corneal nerve was well distributed in both control and NM-fixed corneas without any nerve-degenerated regions (Fig 5).

## Discussion

The nictitating membrane can destabilize contact lenses on the animal eye, and thus be problematic when using animal models to reliably study the effect of new biomaterials on the corneal surface. The nictitating membranectomy is considered an essential procedure for contact lens retention in animal models [10]. However, the procedure is invasive and requires a relatively long surgical time, approximately a week of recovery, and additional surgical procedures such as a cauterization. In addition, the surgery causes excessive tissue damage and presents ethical issues. We therefore explored a new simple, minimally invasive and safe surgical approach that “fixes” the NM in rabbits. The procedure has many merits compared to conventional methods. First, the procedure time for NM fixation was minimal, requiring only the application of a stich between the upper eyelid and the free edges of the nictitating membrane. In addition, NM fixation does not require extensive training or additional instruments other than basic surgical tools. Normal STT values and clinical observations of rabbits after NM fixation demonstrated minimal recovery time. Finally, NM fixation was proven to be a safe procedure that does not induce substantial lesions including corneal inflammation, hyposensitivity, edema and neovascularization (Figs 3 and 5). By avoiding corneal inflammation and hypoxia, vascular endothelial cells do not migrate into the sub-epithelial and stromal space of the cornea, the main etiology of corneal neovascularization that was avoided in the procedure [11]. The safety of the procedure was further strengthened by the buffer effect of the ophthalmic spear that prevents laceration or necrosis of the upper eyelid by excessive tension of the suture knot, one of the most common complications during NM fixation to the upper eyelid [12].

A potential issue of the nictitating membranectomy is the unavoidable excision of the accessory lacrimal gland. Although some animals can compensate for decreased tear production through other lacrimal glands and the harderian gland after removing the NM [13], animal models such as dogs and cats are susceptible to dry eye syndrome [14, 15]. In addition, decreased tear production may affect contact lens retention by reducing hydration of soft contact lenses [7, 16]. Loss of goblet cell clusters in the nictitating membrane could decrease the mucin portion of tears, detracting from tear quality [2]. NM fixation does not require functional tissue manipulation of the NM, including the accessory gland, and a suture could induce only minimal tissue damage on the margin of NM and upper eyelid. We confirmed through STT and TUBT that the NM fixation procedure did not affect the quality and quantity of tears in rabbits. Furthermore, the procedure did not cause any substantial secondary morphological or pathological side effects at 1 month resulting from decreased quality and quantity of tear production.

As shown in the rabbit model, we expect that NM fixation would increase retention of the contact lens on the cornea and be feasible for testing various contact lens–based technologies, such as the drug-releasing contacts for the glaucoma treatment [17]. In addition, this technique could be used in veterinary practice. For instance, the loss of the bandage contact lens on the corneal surface can be an issue when treating animals with chronic corneal epithelial defects, such as dogs [18]. Because NM fixation was performed under the general anesthesia, some issues, such as the anesthetic risk, could be raised in the veterinary practice. However, the procedure could be done with auriculopalpebral nerve block, topical anesthesia of the eye, and infiltration anesthesia as being done in the NM flap [12] that could be reduce issues of the general anesthesia. Besides contact lenses, NM fixation could be used for the application of soft biomaterials, such as fibrin glue, to the animal cornea. The restricted movement of NM may reduce damage to and promote retention of the soft material.

In conclusion, we demonstrated that NM fixation could increase stability of the contact lens without reducing the quality and quantity of tear production in healthy rabbits. In addition, the technique is simple and did not cause any major pathological changes. We suggest NM fixation would be helpful to design animal studies for investigating new contact lens and has potential to apply to other biomaterial research at the ocular surface.
